# Is *Lactobacillus* Gram-Positive? A Case Study of *Lactobacillus iners*

**DOI:** 10.3390/microorganisms8070969

**Published:** 2020-06-29

**Authors:** Hyaekang Kim, Taehyun Kim, Jaeku Kang, Younghoon Kim, Heebal Kim

**Affiliations:** 1 Department of Agricultural Biotechnology and Research Institute of Agriculture and Life Sciences, Seoul National University, Seoul 08826, Korea; hkim458@snu.ac.kr (H.K.); ykeys2584@snu.ac.kr (Y.K.); 2 Department of Obstetrics and Gynecology, Konyang University Hospital, Daejeon 35365, Korea; th2580@kyuh.ac.kr; 3 Priority Research Center, Myunggok Medical Research Institute, College of Medicine, Konyang University, Daejeon 35365, Korea; jaeku@konyang.ac.kr; 4 Department of Pharmacology, College of Medicine, Konyang University, Daejeon 35365, Korea; 5 C & K genomics, H Businesss Park, Seoul 05836, Korea

**Keywords:** *Lactobacillus iners*, TEM, peptidoglycan, gram-negative

## Abstract

*Lactobacillus iners* is the most prevalent bacterial species in the human vaginal microbiome, and there have been few reports of its Gram-negative stain appearances despite the fact that the genus *Lactobacillus* is universally described as Gram-positive. Here, using transmission electron microscopy, we reveal that the thinness of the cell wall (17.39 ± 0.8 nm) gives the Gram-negative stain appearance, which can lead to over-diagnosis of bacterial vaginosis. Moreover, comparative genome analysis identified four genes commonly absent in *L.* *iners* genomes that might contribute to this phenotypic difference. We suggest that, along with the several niche-specific attributes identified, this unique feature may contribute to the species’ distinguished capability to thrive as the predominant species in the fluctuating vaginal environment as well.

## 1. Introduction

*Lactobacillus iners* is the most frequently detected bacterial species in the human vaginal microbiome [1,2,3]. In contrast to the gut microbiota, the healthy human vaginal microbial community is typically characterized by extremely low diversity, and lactobacilli are the representative species [4]. The communities are either dominated by one or a few common *Lactobacillus* sp. (*Lactobacillus crispatus, L. iners, Lactobacillus gasseri*, and *Lactobacillus jensenii*) [2]. These microorganisms protect the host from colonization of potentially pathogenic organisms by producing lactic acids which acidify the vaginal environment to pH 3.5–4.5 or modulation of host immune system [5,6], and when the ecological equilibria are disturbed, the proportion of lactobacilli diminishes and the number of strictly anaerobic bacteria increases, so does the risk of bacterial vaginosis (BV) or other infectious conditions [7,8]. However, unlike other *Lactobacillus* species, *L. iners* is detected in women with healthy conditions as well as with, and recovering from, vaginal dysbiosis [9]. They do not seem to be easily displaced by pathogens or infectious conditions, as *L. iners* could disassemble biofilms of BV-associated pathogens [10], and a recent study proposed that a unique defense system that *L. iners* has might account for a significantly low number of prophages in their genomes, thereby facilitating persistence during BV [11]. They were even reported to be capable of coexisting when infected with *Chlamydia trachomatis* [12,13]. It was also identified that they had an ability to produce inerolysin, a pore-forming protein typically found in pathogenic bacteria, which gives them an enhanced adhesion ability [14]. These characteristics imply that they have been more successfully adapted to the fluctuating vaginal environments than the other vaginal lactobacilli. The gene contents of *L. iners* strains are known to be quite similar, but in health and dysbiosis, they show big differing expression profiles over 10% of their genes, including the inerolysin [15]. They also have several other interesting features that are distinguishable from common *Lactobacillus* species, which might contribute to their adaptive advantages as well, such as the small size of their genome or different nutritional requirements [16,17]. Complete genome sequences of *L. iners* were only recently constructed from clinical isolates of Asian, African American, and Caucasian women, and the genome sizes ranged from 1.3 to 1.4 Mbp, with significantly low GC contents of ~33.3% [11,18]. Only three of the known strains harbor plasmids [18]. The vaginal lactobacilli have an overall tendency towards reduced genome sizes [19], and yet the genome sizes of *L. iners* are as small as to be comparable to those of human symbionts and parasites [17]. In particular, although the genus *Lactobacillus* has been universally described as being Gram-positive, few reports have claimed that *L. iners* strains had Gram-negative stain appearances [20,21]. For this reason, the species has been overlooked in culture and microscopy-dependent approaches until it was first identified in 1999 [22], but the structural or genetic backgrounds of the phenomenon have not been investigated yet. In this study, we aim to identify factors confirming the Gram-negative stain appearance of *L. iners* by uncovering the ultrastructure of its cell envelope using transmission electron microscopy (TEM) and comparative genome analysis with 1192 genomes for nine other *Lactobacillus* species without the controversy of Gram-stained morphology. We expect that the results presented in this work could provide more insights into the niche-specific adaptation of *L. iners*.

## 2. Materials and Methods

### 2.1. Strain Isolation and Growth Condition

For the isolation of *L. iners*, vaginal specimens were provided from two healthy Korean women (34 and 37 years old), who were attending the obstetrics and gynecology outpatient clinic of Konyang University hospital, Daejeon, in South Korea. They showed no symptoms of vaginal infections, as noted by an examining clinician. Vaginal swabs were collected by a clinician after informed written consent was obtained from the participants. This study, including specimen collection, was approved by the ethical review board of Konyang University (IRB FILE No: 2017-12-021-010). The swabs were vigorously agitated in sterile 1 mL of saline to dislodge cells. Each sample was serially diluted ten-fold using sterile saline and 100 μL aliquots from 10^−1^ to 10^−8^ dilution tubes were spread onto Tryptic Soy Agar plates with 5% defibrinated sheep blood (ATCC Broth recipe no. 260). The inoculated plates were incubated anaerobically by using the BD BBL™ GasPack system (Franklin Lakes, NY, USA) for 48 h at 37 °C. The isolation of purified bacteria was performed by picking single colonies and subculturing in Tryptic Soy Broth with 5% defibrinated sheep blood. Catalase-negative bacillus and coccobacillus isolates were determined as presumptive lactobacilli and subjected to 16S rRNA gene sequencing for identification to the species level. *L. plantarum* and *E. coli* OP50, used in comparison, were cultured in de Man-Rogosa-Sharpe (MRS) broth and agar (Difco, Detroit, MI, USA) and Tryptic Soy broth and agar (Difco, Detroit, MI, USA), respectively, and incubated at 37 °C.

### 2.2. 16S rRNA Sequencing for the Identification of L. iners KY

The bacterial strains were sent to Macrogen Inc. (Seoul, Korea) for 16S rRNA sequencing. The following universal primers were used to selectively amplify the bacterial 16S rRNA gene amplicons, and the resulting amplicons were sequenced using ABI 3730xl DNA Analyzer (Applied Biosystems): forward primer 27F (5′-AGA-3′) and reverse primer 1492R (5′-TACGGY-3′). The resulting sequences were compared with sequences in the National Center for Biotechnology Information (NCBI) database using BLAST.

### 2.3. DNA Extraction and Whole Genome Sequencing

Genomic DNA was extracted using PureHelix™ Genomic DNA Prep Kit (Solution Type)-Bacteria (NanoHelix, Daejeon, Korea) with minor modifications. Isolated gDNA was quantified and qualified by gel electrophoresis, 260/280 nm absorbance ratio and Quant-iT™ PicoGreen™ dsDNA Assay Kit (Invitrogen, Carlsbad, CA, USA). The library was prepared using the ONT 1D ligation Sequencing kit (SQK-LSK109) with the native barcoding expansion kit (EXP-NBD104) according to the manufacturer’s protocol. The resulting library was loaded onto the Flongle Flow Cell (FLO-FLG001, R9.4.1) and sequenced on the MinION MK1b and MinKNOW (19.06.8).

### 2.4. Genome Assembly and Annotation

Base calling and de-multiplexing were carried out using Guppy (v3.4.3), and Porechop (v0.2.4, https://github.com/w1bw/Porechop) was employed to remove the sequencing artifacts and chimeric reads. Genome assembly was conducted using CANU (v1.8) [23] with genomeSize = 1.3 Mb parameter, and for assembly polishing, Nanopolish (v0.11.1) [24] was repeatedly applied using Illumina reads generated on an Illumina iSeq 100 until no correction is available to increase consensus accuracy. Genome annotation was conducted using the Prokka (v1.14.5) [25] with-rfam option to enable a search for ncRNA. In addition, automatic annotation results were collected from Rapid Annotation using Subsystem Technology (RAST) [26].

### 2.5. Comparative Genome Analysis

For comparative genome analysis, 1192 available genomes for 10 *Lactobacillus* species in the NCBI database were used as of 18 March 2020. In total, 10 *Lactobacillus* species consisting of *L. iners* and 9 other species were analyzed without the controversy of Gram-stained morphology (*L. platarum*, *L. acidophilus*, *L. crispatus*, *L. gasseri*, *L. jensenii*, *L. helveticus*, *L. johnsonii*, *L. reuteri*, *L. salivarius*). First, 4 genomes randomly selected from each species were used to construct the gene clusters using OrthoMCL [27] after annotation. Then, we identified gene clusters commonly absent in *L. iners* genomes but conserved in other *Lactobacillus* genomes, and further filtering with the rest of the genomes based on BLASTP was conducted, since OrthoMCL cannot handle the 1192 whole genomes used in this study.

### 2.6. Transmission Electron Microscopy (TEM) and Gram-Staining Test

Sample processing and TEM analysis were performed at the Nanobio imaging center of the National Instrumentation Center for Environmental Management (NICEM), Seoul National University, Korea. For the preparation of TEM samples, *L. iners* KY and *L. plantarum* cells were grown to mid-exponential growth phase and centrifuged at 4000 rpm for 10 min at 4 °C. Sample processing was done as follows: cell pellets of each strains were fixed with Karnovsky’s fixative, post-fixed with 1% osmium tetroxide in 0.1 M sodium cacodylate buffer for 2 h, and then stained with 0.5% uranyl acetate overnight at 4 °C. After dehydration in a graded ethanol series (50, 70, 80, 90, and 100%), they were embedded in Spurr’s resin. The samples were sectioned at 70 nm using an ultra-microtome (EM UC7; Leica Microsystems, Vienna, Austria), stained with 2% uranyl acetate and Reynolds’ lead citrate. Then, they were visualized using transmission electron microscope Talos L120C (FEI, Czech Republic) operated at 120 kV. For each strain, cell wall thickness measurements were taken from 12 intact cells. Gram-staining was performed using the BD BBL™ Gram staining kit following the manufacturer’s protocol.

## 3. Results and Discussion

**Figure 1:**
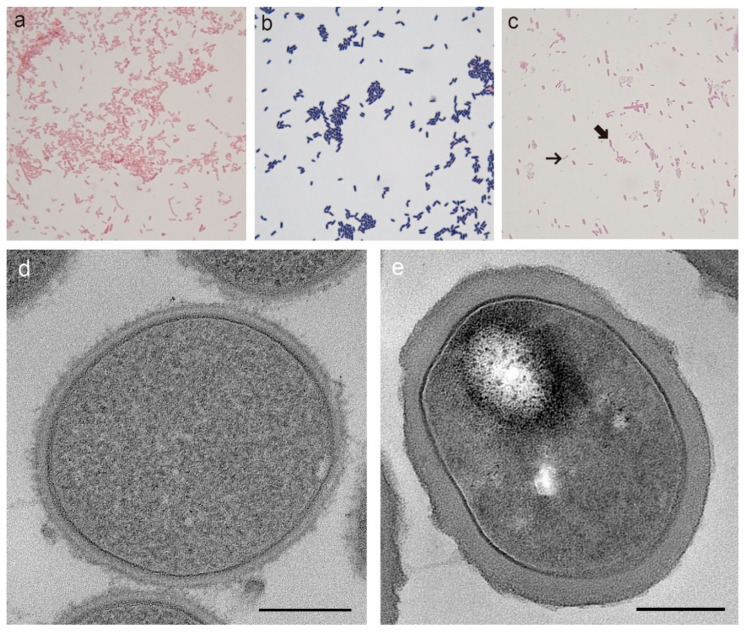
Microscopic images of strains. (**a**–**c**) Gram-stains of (**a**) *L. iners* KY. (**b**) *L. plantarum*. (**c**) artificial mixtures of *L. iners* KY (narrow arrow) and *E. coli* OP50 (wide arrow). (**d**,**e**) TEM images of cell envelopes of (**d**) *L. iners* KY. (**e**) *L. plantarum*. Both species were identically processed. It is shown that *L. iners* has the cell envelope organization of the Gram-positive organisms, but the thickness of the peptidoglycan layer is relatively thin compared to *L. plantarum*. Scale bar = 200 nm.

We isolated the *L. iners* strain KY from the vaginal swabs of a 34-year-old woman with healthy, asymptomatic vaginal profiles and confirmed using 16S rRNA sequencing. First, we carried out Gram-staining of the strain, and all of the cells were clearly stained Gram-negative (Figure 1a). They were also confirmed as Gram-negative when compared to *Lactobacillus plantarum* (Figure 1b), and when the strain was stained with *Escherichia coli* OP50 in the artificial mixtures, their stained appearances were hardly distinguishable from each other (Figure 1c). To further examine the ultrastructure of its cell wall, transmission electron microscopy (TEM) of sectioned cells was performed on the *L. iners* KY and *L. plantarum*. Since it has been noted that both TEM and Gram-staining are affected by aging [28,29], we used cells in mid-exponential growth phases. TEM images clearly resolved cell envelopes, consisting of the plasma membrane and peptidoglycan (PG) layer (Figure 1d,e). A higher magnification of an individual cell revealed that the PG layer of *L. iners* was very thin, and the thickness was approximately one-third of that in *L. plantarum* (Figure 1d,e). The size of the *L. iners* PG layer was 17.39 ± 0.8-nm thick, when averaged from 12 measurements of morphologically intact cells, which is rather thin for typical Gram-positive organisms (20–80 nm) [30]. In previous studies, *Gardnerella vaginalis* had also been described to have a small thickness (8–12 nm) for the PG layer, within a classic Gram-positive wall architecture, and it stained negatively as well [31]. *L. iners* is the first *Lactobacillus* species reported by far whose PG layer is thin enough to give apparent Gram-negative morphologies. This finding challenges the common understanding that all organisms of genus *Lactobacillus* stain Gram-positive. This structural characteristic and staining property of *L. iners* are clinically very important to consider since the Nugent scoring remains a common diagnostic tool to assess vaginal health [16]. This test presumes all lactobacilli as Gram-positive rods and BV-associated pathogens as Gram-negative, with Gram-variable rods and cocci. Furthermore, given that *L. iners* is the most prevalent species, this can lead to the false interpretation of the vaginal smear [7]. In fact, it has been described that as many as 50% of women diagnosed with BV using the Nugent score were asymptomatic [32].

Whole-genome sequencing of *L. iners* KY was performed using the MinION platform (Oxford Nanopore Technologies), and the first complete genome of *L. iners* species was constructed in our previous study [11]. Analysis of its genome revealed that it had a severely reduced number of genes related to carbohydrate and amino acid metabolism, compared to other lactobacilli (Table 1). *L. iners* is the smallest genome among lactobacilli determined by far (~1.3 Mbp) as a result of massive genome reduction events [17]. Despite its global loss in gene contents, it maintained a substantial proportion of membrane transport genes, which indicates that it may have become, in its evolutionary path, largely dependent on the utilization of essential compounds from the hosts or the community (Table 1). With this unique thin PG layer, *L. iners* cell envelopes may be more permeable for nutrient uptake or protein secretion than other lactobacilli, thereby facilitating the replenishment of numbers or effectively responding to changes in the nutritional source in their environment. To determine whether related genes were lost as a result of genome reduction, the distribution of genes involved in the PG metabolism was compared in genomes of 40 *Lactobacillus* strains (four randomly selected from each ten *Lactobacillus* species including *L. iners* KY), and it was shown that *L. iners* was capable of encoding all the enzymes directly involved in the PG synthesis and hydrolysis (Table S1). We, therefore, performed further investigation to search gene clusters commonly absent from *L. iners* genomes that were conserved in other lactobacilli by defining gene families using OrthoMCL [27] analysis with 1192 *Lactobacillus* genomes (all available genomes of those ten *Lactobacillus* species), and four genes (*csbB*, *xynA*, *yvgN*, *phoU*) were identified to be absent only in *L. iners* genomes (Table 2). Among them, CsbB is a membrane-bound protein that acts as a bactoprenol (undecaprenyl phosphate) glycosyltransferase. It was demonstrated in *Bacillus subtilis* that this protein was involved in the lipoteichoic acid sugar modification with *N*-acetylglucosamine [33]. Moreover, bactoprenol itself is a key molecule of the PG synthesis in which it translocates the newly synthesized PG monomers to the outside surface of the membrane, where they are inserted to the PG matrix [34]. Although no additional functional roles have been investigated yet, our comparative genome analysis results suggest a potential role of CsbB in the regulation of PG metabolism in an as yet unknown mechanism. Surprisingly, we found that *csbB* was also absent in all available *G. vaginalis* genomes that give similar Gram-stain results due to the thin PG layer. Future biochemical studies are required to better understand its functional interactions with other components of PG metabolism. Our study identified that the only possible factor that can account for the Gram-negative stain characteristic of *L. iners* was their thin PG layer and presented candidate genes that may functionally contribute to the phenotypic characteristics. Moreover, we suggest that, along with several niche-specific genes already identified, this unique feature of *L. iners* may provide an adaptive advantage to thrive as the predominant species in the vaginal environment as well.

## Figures and Tables

**Table 1:** Functional categories of genes in genomes of *L. plantarum* WCFS1, *L. crispatus* ST1, *L. iners* KY.

Functional Category/Pathway	Number of Genes		
	*L. plantarum* WCFS1	*L. crispatus* ST1	*L. iners* KY
Carbohydrate metabolism	258	85	35
Amino acid metabolism	189	80	22
Lipid metabolism	39	25	22
Nucleic acid metabolism	89	69	61
Cofactor metabolism	104	51	40
Membrane transport	49	21	19
Replication & repair	50	38	35
Transcription	20	15	14
Translation	116	109	109
Cell Wall	76	43	20
Stress Response	20	3	6
Phages, Prophages, Transposable elements, Plasmids	10	0	5
Virulence, Disease and Defense	38	37	15
CRISPRs	0	6	2

**Table 2:** Proteins commonly absent in *L. iners* genomes that are conserved in nine *Lactobacillus* genomes.

Protein	Function
XynA	Alpha/beta hydrolase
CsbB	Putative glycosyltransferase CsbB
YvgN	Glyoxal reductase
PhoU	Phosphate signaling complex protein PhoU

## Supplementary Materials

The following are available online at https://www.mdpi.com/2076-2607/8/7/969/s1, Table S1: Distribution of genes encoding enzymes involved in the peptidoglycan metabolism in 40 *Lactobacillus* strains.
